# Treatment of Common Sunflower (*Helianthus annus* L.) Seeds with Radio-frequency Electromagnetic Field and Cold Plasma Induces Changes in Seed Phytohormone Balance, Seedling Development and Leaf Protein Expression

**DOI:** 10.1038/s41598-019-42893-5

**Published:** 2019-04-23

**Authors:** Vida Mildažienė, Vesta Aleknavičiūtė, Rasa Žūkienė, Giedrė Paužaitė, Zita Naučienė, Irina Filatova, Veronika Lyushkevich, Perttu Haimi, Inga Tamošiūnė, Danas Baniulis

**Affiliations:** 1 Faculty of Natural Sciences, Vytautas Magnus University, Kaunas, Lithuania; 2 B. I. Stepanov Institute of Physics, National Academy of Sciences of Belarus, Minsk, Belarus; 3 Institute of Horticulture, Lithuanian Research Centre for Agriculture and Forestry, Babtai, Kaunas reg, Lithuania

**Keywords:** Biochemistry, Plant sciences

## Abstract

Treatment of plant seeds with electromagnetic fields or non-thermal plasmas aims to take advantage of plant functional plasticity towards stimulation of plant agricultural performance. In this study, the effects of pre-sowing seed treatment using 200 Pa vacuum (7 min), 5.28 MHz radio-frequency cold plasma (CP −2, 5, and 7 min) and electromagnetic field (EMF −5, 10, 15 min) on seed germination kinetics, content of phytohormones, morphometric parameters of seedlings and leaf proteome were assessed. CP 7 min and EMF 15 min treatments caused 19–24% faster germination *in vitro*; germination in the substrate was accelerated by vacuum (9%) and EMF 15 min (17%). The stressors did not change the seed germination percentage, with exception of EMF 5 min treatment that caused a decrease by 7.5%. Meanwhile both CP 7 min and EMF 15 min treatments stimulated germination, but the EMF treatment resulted in higher weight of leaves. Stressor-specific changes in phytohormone balance were detected in seeds: vacuum treatment decreased zeatin amount by 39%; CP treatments substantially increased gibberellin content, but other effects strongly varied with the treatment duration; the abscisic acid content was reduced by 55–60% after the EMF treatment. Analysis of the proteome showed that short exposure of seeds to the EMF or CP induced a similar long-term effect on gene expression in leaves, mostly stimulating expression of proteins involved in photosynthetic processes and their regulation.

## Introduction

An interdisciplinary field of research on low temperature non-equilibrium plasma, also termed cold plasma (CP) and electromagnetic field (EMF) applications for agriculture^1,2^ is directed towards exploiting the potential of plant functional plasticity. Seed treatment with CP or EMF is a modern eco-agricultural technology for increasing plant agricultural performance. Numerous studies have demonstrated that such treatments are effective for enhancing agronomic seed quality and have potential to be used for seed decontamination, activation of germination and seedling growth.

The majority of studies in this area are focused on assessment of CP and EMF effects on physiological (germination), structural (changes in seed coat surface) and morphometric (early seedling growth) estimates (reviewed by^3–5^). A few reports have also considered changes in biochemical characteristics, such as amount of pigments and secondary metabolites, enzymatic activities or antioxidative capacity^6–12^. Stressor-induced changes in physiological or biochemical activities are associated with selective modulation of protein expression in the growing seedling, e.g., activation of photosynthesis is expected to be related to changes in the leaf proteome^13,14^. Although several studies described morphological, genotoxic or biochemical changes induced by the CP and EMF treatments^14–19^, the proteomic profiles of plant response to CP and EMF treatments have not been reported so far.

On the other hand, although germination tests are commonly used as a hallmark of the response to treatments, until now there has been no attempt to estimate CP and EMF effects on the content of seed phytohormones, which are known to be key regulators of germination^20^. Plant hormones regulate seed dormancy and germination through an integrated network of interactions where the primary role belongs to an antagonistically acting duo: inhibitor of germination abscisic acid (ABA) and stimulators of germination gibberellins (GAs)^21,22^. Numerous other hormones exert impact on germination by modulating the effects of ABA/GA balance: auxin IAA (indole-3-acetic acid) is known to be a negative regulator of germination; ethylene, citokinins, brasinosteroids, and strigolactones can stimulate germination by various modes; salicylic acid (SA) and jasmonate (stress hormones) may affect germination positively or negatively depending on the situation^20^.

Our study is aimed to gain insight into the molecular mechanisms underlying the effect of physical stressors (vacuum, CP and EMF) on plants. The effects of pre-sowing seed treatments on phytohormone content in seeds, germination kinetics and growth of the seedlings of the common sunflower (*Helianthus annuus* L.) has been assessed. This plant is often used for seed physiology studies^23^ and is characterized by the physiological (controled by phytohormones) dormancy^24^. It became a model plant species for numerous eco-physiological studies due to the economic importance, available information on genome sequences and transcriptomic data^25^. Bearing in mind that germination is affected by a mutually interactive network of phytohormones, we have estimated the effects of vacuum, CP and EMF treatments on the amount of ABA, GA, auxins IAA and IBA (indole-3-butyric acid), citokinin zeatine (Z), and SA in dry seeds. In addition, changes in protein expression patterns in leaves and roots of sunflower seedlings have been determined. The study has revealed that the effects of CP and EMF treatments on seed germination are related to changes in phytohormone content, and the effects on seedling growth mostly have been related to differences in photosynthetic machinery protein expression.

## Results

### Changes in sunflower germination kinetics and seedling morphology induced by seed treatment with a vacuum, CP and EMF

**Figure 1:**
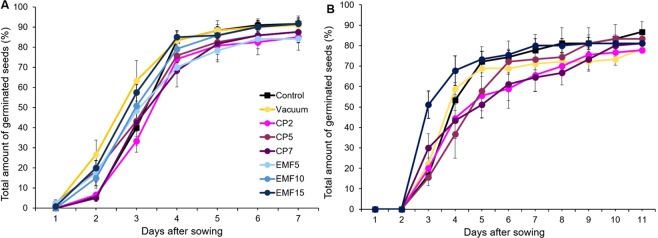
Germination dynamics of sunflower seeds *in vitro* (**A**) and in substrate (**B**). The points represent mean values of three replicates ± standard error of mean. Seed treatments for all experimental conditions were replicated three times (n = 30 for one replicate).

The performed germination tests showed that pre-sowing treatment of sunflower seeds with vacuum, CP and EMF induced changes in both germination kinetics *in vitro* (Fig. 1A) and in the substrate (Fig. 1B), and these changes depend on the treatment duration and germination conditions.

Analysis of the germination curves (Fig. 1) using Richards plots and calculated germination indices were used to quantitate the observed changes (Table 1). None of the used seed treatments affected the germination yield or final germination percentage (Vi), except CP5 treatment that slightly (by 7.5%) decreased Vi for germination *in vitro*. The median germination time (Me) *in vitro* decreased in the groups of seeds treated with CP7, EMF10 and EMF15 by 20, 24 and 19%, respectively, indicating that the germination rate was enhanced *in vitro*. However, when seeds germinated in the substrate, CP7 and EMF10 treatments were not effective, instead Me was slightly smaller (by 7%) in the vacuum treated group. Only the EMF15 treatment increased germination rate by 16% in comparison to the control, similarly to the germination *in vitro* (Table 1).

**Table 1:** Indices of germination kinetics of sunflower seeds derived from Richards plots.

Treatment	Germination *in vitro*		Germination in substrate	
	Vi,%	M_e_, days	Vi,%	M_e_, days
Control	91.7 ± 2.2	3.52 ± 0.21	86.7 ± 5.1	4.7 ± 0.1
Vacuum	90.8 ± 4.6	3.03 ± 0.15	78.9 ± 1.1	4.3 ± 0.1^*^
CP2	85.0 ± 6.5	3.15 ± 0.10	77.8 ± 1.1	5.2 ± 0.8
CP5	87.5 ± 5.2	3.57 ± 0.17^**^	83.3 ± 1.9	5.2 ± 0.4
CP7	87.5 ± 2.5	2.82 ± 0.27^*^,^**^	81.1 ± 2.2	5.2 ± 0.6
EMF5	84.2 ± 2.1^*^	3.65 ± 0.31	—	—
EMF10	91.7 ± 1.7	2.67 ± 0.27^*^	—	—
EMF15	91.7 ± 2.9	2.87 ± 0.25^*^	81.1 ± 2.9	3.9 ± 0.2^*^

Mean values ± standard error of mean are presented (n = 3 replicates, 30 seeds in one replicate), ^*^significantly different from the control group (p ≤ 0.05); ^**^significantly different from the vacuum group (p ≤ 0.05).

After germination in the substrate sunflower seedlings were grown for two weeks and the effects of pre-sowing sunflower seed treatment on early seedling growth were estimated (Table 2). The results of morphometric seedling analysis revealed negative effects of CP7 treatment on early seedling development – compared to the control, length and weight, shoot length and weight of seedlings derived from CP7 treated seeds was smaller by 19, 15, 14 and 14%, respectively. Thus, early growth of CP7 seedlings was obviously suppressed despite the stimulation of germination *in vitro* (Table 1). Negative effects of vacuum and CP2 treatments on seedling growth were observed as well, but only as a reduction in seedling length by 11 and 13%, respectively. The only positive effect of seed treatments was 14% increased weight of leaves in EMF15 group. EMF15 seedlings did not differ from the control seedlings by any other morphometric parameters. Thus, seedlings from EMF15 group exhibited the most positive response and those from CP7 group – the most negative response to seed treatment at the stage of early growth. To further assess the molecular basis of the effects, seedlings from the CP7, EMF 15, vacuum and control groups were selected for leaf proteome analysis.

**Table 2:** Morphometric parameters of sunflower seedlings 2 weeks after sowing.

Treatment	Seedling		Roots		Shoot		Leaves
	Length, cm	Weight, g	Length, cm	Weight, g	Length, cm	Weight, g	Weight, g
Control	21.6 ± 1.0	1.36 ± 0,06	9.8 ± 0.9	0.13 ± 0.03	11.7 ± 0.6	1.23 ± 0.06	0.70 ± 0.03
Vacuum	19.1 ± 0.9^*^	1.37 ± 0.07	8.5 ± 1.0	0.12 ± 0.01	10.8 ± 0.5	1.25 ± 0.07	0.74 ± 0.04
CP2	18.9 ± 0.9^*^	1.40 ± 0.10	7.9 ± 1.0	0.12 ± 0.01	11.0 ± 0.5	1.28 ± 0.09	0.75 ± 0.06
CP5	19.4 ± 1.0	1.32 ± 0.68	8.7 ± 1.1	0.13 ± 0.01	10.7 ± 0.7	1.20 ± 0.06	0.70 ± 0.05
CP7	17.6 ± 0.9^*^	1.15 ± 0.08^*^	7.9 ± 0.9	0.09 ± 0.01	10.1 ± 0.4^*^	1.06 ± 0.07^*^	0.65 ± 0.06
EMF15	21.0 ± 0.1	1.46 ± 0.08	9.9 ± 1.2	0.11 ± 0.01	10.8 ± 0.8	1.35 ± 0.08	0.80 ± 0.05^*^

Mean values ± standard error are presented (n = 17–24 seedlings), ^*^significantly different from the control group (p ≤ 0.05).

### Changes in phytohormone content induced by seed treatment with vacuum, CP and EMF

The results of phytohormone analysis performed four days after seed treatment revealed significant changes in phytohormone content that were induced by the pre-sowing treatments of sunflower seeds. Vacuum treatment did not induce changes in seed ABA, GA, and SA content but significantly changed the auxin/cytokinin balance – in vacuum treated seeds, IAA dropped down below a detectable level, IBA amount increased more than 4 times and Z amount decreased by 39%. As a result (IAA + BA)/Z ratio decreased more than 5 times, compared to the control. All CP treatments substantially increased GA3 while decrease in Z amount was similar to that of the vacuum treatment. Effects on content of other hormones varied and were dependent on seed treatment duration. E.g., ABA amount increased in CP2, decreased in CP5 and did not change in CP7 group, while only CP7 treatment elicited increase in seed IAA content. All durations of EMF treatment reduced the amount of ABA in sunflower seeds by more than 50%, substantially increased IAA (4.4–5.4 fold) and SA (35–50 fold) but did not change GA, IBA and Z content.

### Differential protein expression in sunflower seedlings and protein function analysis

**Figure 2:**
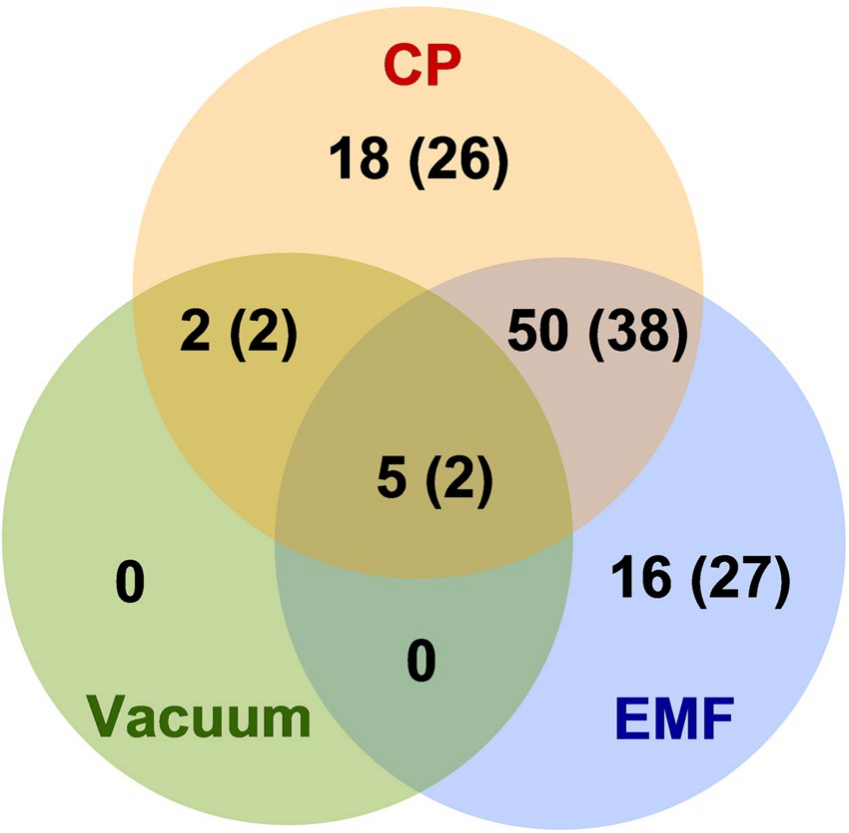
Venn diagram of protein abundance differences (p < 0.01 and >1.5-fold variations) in the sunflower shoots germinated from seeds treated with vacuum, CP or EMF radiation. Numbers outside and inside brackets indicate differences when the treated experimental group is compared to untreated control or vacuum treated experimental groups, respectively.

Proteomics analysis was used to assess differential protein expression of sunflower seedlings germinated from the seeds treated with vacuum, CP or EMF. Since a narrow isoelectric point (pI) range and protein solubility would be preferable for efficient protein separation via 2D electrophoresis, the acidic range of pH 4–7, corresponding to dominant pI value of cytosolic proteins^26,27^, was selected for fractionation using isoelectrical focusing. After gel alignment, the average number of detected protein spots was 1910 ± 327 per gel (Supporting Material Fig. S1). Among the four experimental groups (control, vacuum, CP or EMF treatment), 104 proteoforms had statistically significant (p < 0.01) and > 1.5-fold variations in abundance in shoot samples (Fig. 2). Meanwhile all differences among the experimental groups in the root samples were below the significance threshold. Vacuum treatment had no specific effect on sunflower seedling proteome.

Through liquid chromatography – tandem mass spectrometry (LC-MS/MS) fingerprinting of trypsin digested peptides, 41 proteoforms differentially expressed in shoots were unequivocally identified, corresponding to 33 unique sunflower proteins (data are shown in Supporting material Table S1).

**Figure 3:**
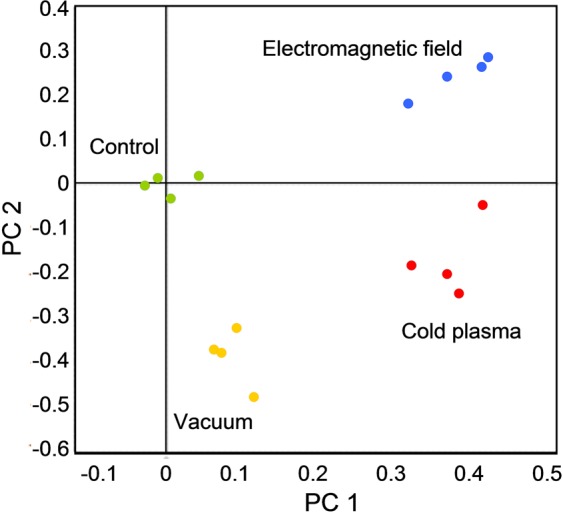
Principal component analysis of the differentially expressed protein data in sunflower shoots germinated from the seeds treated with vacuum and/or CP and EMF. Spots of the same color represent four biological replicates.

An assessment of the relationship among the experimental groups by principal component analysis revealed that the first two principal components clearly differ among all four groups (Fig. 3). The largest variance, represented by the first component, was observed between the control and vacuum treatment groups and the two CP or EMF treatment groups. Additional differences between the control and vacuum treatment groups and between the CP or EMF treatment groups were revealed by the second component.

**Figure 4:**
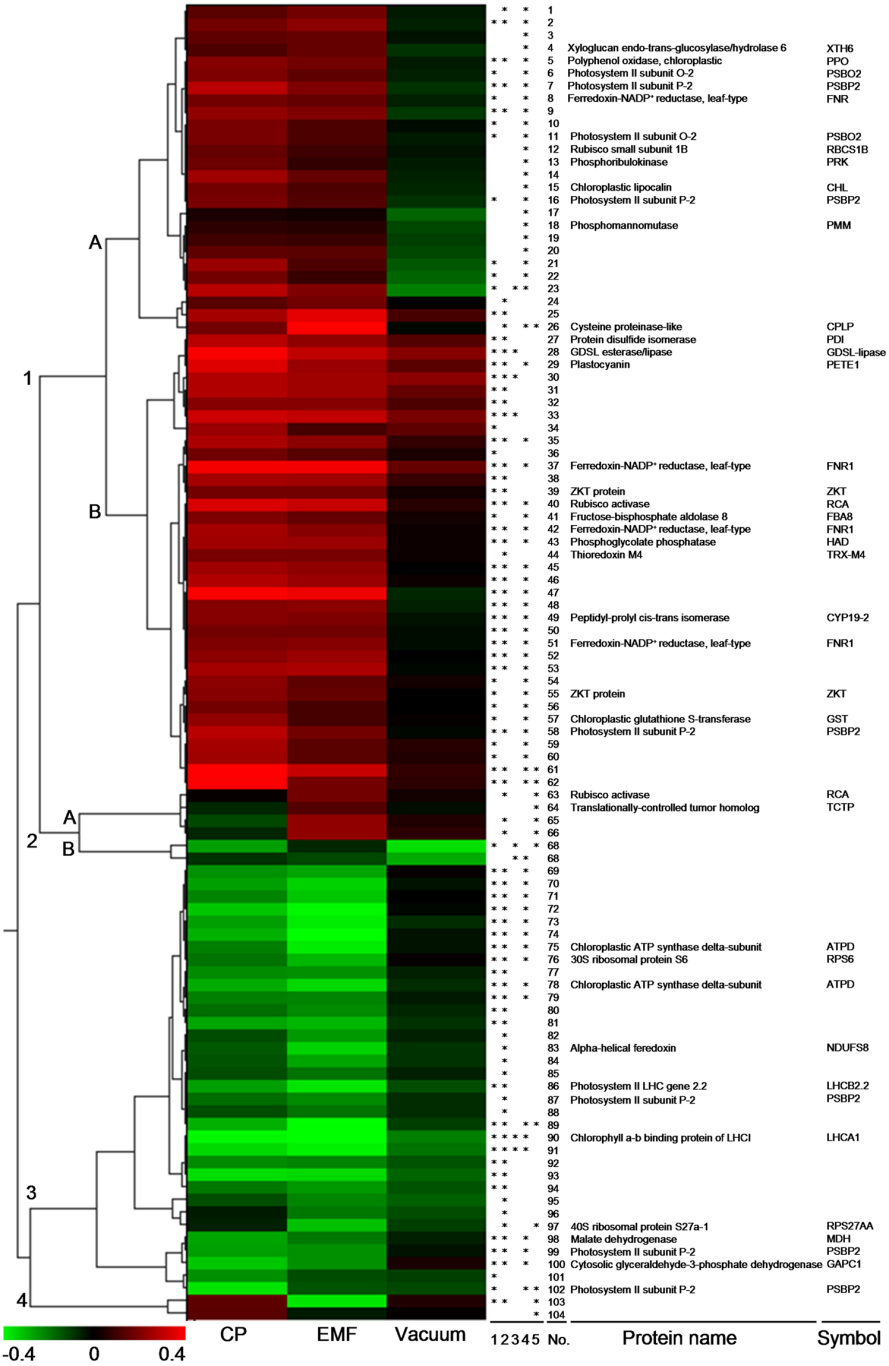
Hierarchical cluster analysis results of the abundance data of proteoforms differentially expressed in sunflower shoots germinated from the seeds treated with vacuum, CP or EMF radiation. Numbers on the left indicate four major clusters based on expression patterns. Colors indicate a decrease (green) or increase (red) in protein abundance compared to control. Star symbols in columns 1–5 indicate statistically significant (p < 0.01) differences between the cold plasma treatment and control (1), electromagnetic field treatment and control (2), vacuum treatment and control (3), cold plasma and vacuum treatment (4), and cold plasma and electromagnetic field treatment (5). Spot number and protein name are shown in column 6.

A hierarchical cluster analysis of protein abundances revealed several distinct expression patterns among the 104 differentially expressed proteoforms (Fig. 4). The largest group (Fig. 4, set 1) included 62 proteoforms that were upregulated by the CP or EMF treatment as compared to the control and/or vacuum treatment. This group could be further divided into two smaller subsets. The first subset (1A) includes 26 proteoforms with lower overall protein abundance differences (from 1.6 to 2.4-fold) within the protein group. Meanwhile, statistically significant differences were mostly observed for the CP treatment as compared to the vacuum treatment. The proteoforms of the subset 1B were upregulated to similar extent by both treatments. Another large cluster included 34 proteoforms that were downregulated upon the CP/EMF treatment (Fig. 4, set 3). The EMF-induced differences were larger compared to the differences observed upon the CP treatment in this cluster. The remaining 8 differentially expressed proteoforms mostly represented differences among the treatment experimental groups and were assigned to two separate clusters (Fig. 4, set 2 and 4). The identified proteoforms of chloroplastic proteins, rubisco activase (RCA) and translationally-controlled tumor homolog (TCTP), were among the four protein spots (subset 2A) that were specifically upregulated by the EMF treatment.

Biological processes were assigned based on gene ontology (GO) data of the identified proteins and the summary of the GO terms of biological process provided in Supporting Information Fig. S2. For 2 of the identified proteins of the set 2, no biological process GO terms were assigned. Twenty-three unique proteins included in the first set were associated with 92 GO terms that were summarized as 22 distinct biological processes based on semantic similarity (Fig. S2, panel A). The proteins were related to cell metabolism (56.1% frequency), and their more specific function was related to broad range of processes that included oxidation-reduction (7.5%), proteolysis (5.5%), carbohydrate metabolism (4.7%), response to cold (1.6%) and photosynthesis (1.1%) and other lower frequency processes mostly related to carbon fixation and utilization or metabolism of biological molecules (fructose, GDP-mannose, glycerol ether, glyoxalate, inositol, tyrosine, pigments). Nine proteins that were identified in the set 3 were associated with 35 GO terms and summarized as 8 distinct and rather unique processes related to oxidation-reduction (7.5%), carbohydrate metabolism (4.5%), translation (3.3%), ribosome biogenesis (2.1%), photosynthesis (1.1%), glucose (0.2%) and glyoxylate (0.04%) metabolism (Fig. S2, panel B).

**Figure 5:**
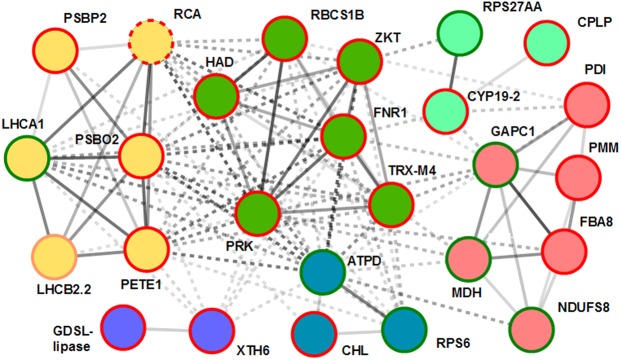
A protein interaction network using *A. thaliana* proteins most closely related to the proteins (groups 1 and 4) that were differentially expressed in sunflower shoots germinated from the seeds treated with vacuum, CP or EMF radiation. The protein interaction network was built using the String database. Circles connecting solid and dashed lines indicate protein interactions within and between clusters, respectively. Circle colors represent protein clusters assigned based on the protein interaction data. Circle line color represents a decrease (green), increase (red) or contrasting regulation of protein abundance for different proteoforms (orange) compared to control. Dashed circle line indicates regulation specific to the EMF treatment.

To assess interactions among the identified proteins, 33 homologous proteins of *A. thaliana* were queried into the String database. The results revealed a network of six closely interlinked interaction clusters centered around proteins that were mainly involved in energy metabolism (photosynthesis, glycolysis) and protein metabolism (Fig. 5). Two interaction clusters (circled in green and purple) consisted exclusively of the proteins that increased in abundance upon the CP/EMF treatment, and the remaining clusters included proteins that had contrasting expression regulation in response to the seed treatment.

The core of the protein network (circled in green in Fig. 5) includes enzymes involved in Calvin cycle reactions (rubisco small subunit 1B (RBCS1B), phosphoribulokinase (PRK), phosphoglycolate phosphatase (HAD)), proteins directly involved in photosynthetic electron transfer and regulation of the linear and cyclic electron flow (ferredoxin-NADP^+^ reductase (FNR1), thioredoxin M4 (TRX-M4)^28^), as well as the regulatory ZKT protein that was proposed to act as a molecular adaptor in chloroplasts, relaying information in signal transduction pathways^29^. Several proteins in the closely interlinked cluster (circled in yellow) were also related to photosynthetic electron transfer activity in chloroplasts and included the chlorophyll a-b binding protein (LHCA1) and subunit 2.2 (LHCB2.2) of the light harvesting complexes I and II, O-2 (PSBO2), P-2 (PSBP2) and rubisco activase (RCA) subunits of the photosystem II, as well as the lumenal electron carrier plastocyanin (PETE1).

Functional or gene expression data linked the majority of the remaining protein interaction clusters to the described network of proteins involved in the photosynthetic apparatus. Three chloroplastic proteins (blue circles) are directly linked to photosynthetic process such as ATP synthase delta-subunit (ATPD)^30^, chloroplastic lipocalin (CHL)^31^, and the 30S ribosomal S6 (RPS6) protein^32,33^. A function of several proteins responsive to the CP/EMF-treatment is localized mainly to the cytosolic compartment and is involved in protein synthesis and gene expression regulation that often has wider implications including modulation of photosynthetic energy transfer and carbon metabolic processes. These included cytosolic glyceraldehyde-3-phosphate dehydrogenase 1 (GAPC1) and fructose-bisphosphate aldolase (FBA1)^34–36^, phosphomannomutase (PMM)^37^, ribosomal protein S27A (RPS27AA)^38^, peptidyl-prolyl cis-trans isomerase (CYP19–2)^39^ and protein disulfide isomerase (PDIL1–2)^40,41^.

## Discussion

Radio-frequency low-pressure plasma or CP is a complex stressor and its different components including high frequency electromagnetic radiation, UV radiation, vacuum, charged particles and subsequently formed reactive chemical species could have an impact on biological systems^2,42^. In comparison to CP, vacuum and EMF are less complex physical stressors, but their effectiveness in inducing changes in germination and plant growth is comparable^1,16^. However, the detailed mechanisms of action are poorly established^3^.

In comparison to the positive effects of sunflower seed treatments with a static magnetic field reported earlier^43^, we revealed rather moderate effects of seed treatment with vacuum, CP and EMF on germination and early growth of sunflower. The comparison of the effects on germination *in vitro* and germination in the substrate revealed a dependence of the observed effects on conditions of seed germination. EMF15 treatment caused faster germination under both conditions; vacuum induced a similar effect only for germination in the substrate, whereas CP7 only stimulated germination *in vitro*. Similar differences have been previously detected in germination of the Norway spruce seeds^12^. That could be explained in several ways. Germination *in vitro* reflects an early or *sensu stricto* germination^44^ stage, whereas germination in the substrate is linked to a later stage of germination. Slower penetration of water, reduced oxygen and light supply^45^, presence of various compounds in the substrate or interaction with microorganisms may also cause differences of seed germination in the substrate compared to that in a Petri dish.

Changes in seed phytohormone content induced by a short (2–15 min) treatment with vacuum, CP and EMF were estimated for the first time. Contrary to the common expectation for exceptional seed stress resistance, the obtained results demonstrated that short pre-sowing treatment of sunflower seeds resulted in significant shift in phytohormone balance. Moreover, the pattern of the induced changes was obviously stressor-specific. Vacuum treatment affected auxin/cytokinin balance; CP treatments substantially increased GA amount, while the other effects varied strongly with the treatment duration; EMF treatments decreased the amount of ABA and increased IAA and SA levels without changes in GA, IBA and Z content. However, the relationship between changes in phytohormone amount (Table 3) and germination kinetics (Table 1) was not straightforward. For example, the stimulation of germination in the substrate by vacuum (for germination *in vitro* the effect was not statistically significant) may be related to a decrease in IAA content. Despite the decreased ABA and increased GA3 amount, germination of CP5 seeds was not stimulated. Although changes in phytohormone balance were similar for all EMF treated seeds, the germination yield *in vitro* decreased in the EMF5 group, and the germination rate increased only in EMF10 and EMF15 groups. This indicates that a certain important part of the information about key hormonal determinants is still missing, possibly because a limited selection of phytohormones that are involved in seed germination has been used. Since phytohormones function in a complex network involving mutual regulation or functional cross-talk^13^, an integrated phytohormone analysis is required to better understand the obtained results.

**Table 3:** The amount of phytohormones in sunflower seeds four days after treatment.

Treatment	Seed phytohormone amount, mean concentration ± SEM (µg/g seed weight)					
	ABA	GA3#	IAA	IBA	Z	SA
Control	2.0 ± 0.5	ND	13.7 ± 1.8	0.4 ± 0.1	4.1 ± 0.7	0.1 ± 0.0
Vacuum	1.8 ± 0.2	ND	ND	1.7 ± 0.1^*^	2.5 ± 0.6^*^	ND
CP2	3.3 ± 0.4^*^	6.0 ± 0.5^*^	ND	0.2 ± 0.1	2.1 ± 0.4^*^	ND
CP5	1.0 ± 0.2^*^	14.0 ± 2.2^*^	ND	1.1 ± 0.2	1.2 ± 0.6^*^	1.3 ± 0.6^*^
CP7	2.2 ± 0.2	19.7 ± 4.1^*^	27.9 ± 3.2^*^	0.5 ± 0.1	2.7 ± 0.3^*^	ND
EMF5	0.8 ± 0.1^*^	ND	60.5 ± 11.2^*^	0.7 ± 0.2	4.3 ± 0.6	4.0 ± 0.5^*^
EMF10	0.8 ± 0.3^*^	ND	66.4 ± 6.2^*^	0.5 ± 0.1	4.1 ± 1.1	5.0 ± 0.7^*^
EMF15	0.9 ± 0.1^*^	ND	73.3 ± 3.1^*^	0.5 ± 0.1	3.2 ± 0.9	3.5 ± 0.5^*^

^#^The amount GA7 was below detectable level in seed of all experimental groups. ^*^Significantly different from the control group (p ≤ 0.05).

The effects of seed treatments on germination did not directly correlate with the changes induced in early seedling growth. Although CP7 increased germination rate *in vitro*, it affected morphometric growth parameters negatively. Similarly, vacuum reduced germination half-time in the substrate but slightly decreased seedling length. Only EMF15 treatment resulted in higher rate of germination and an increase in leaf weight, the rest of the morphometric indices did not differ from the control. Leaf proteome analysis was performed aiming to link the induced changes in seedling growth with a pattern of changes in protein expression levels.

Sunflower seedling proteome analysis showed that vacuum treatment of sunflower seeds had no specific effect on protein abundance in germinated plants (Fig. 2). This implies that upon CP treatment, gene expression differences were more likely to be specifically induced by exposure to radiation or chemical components of the CP. Furthermore, the seed treatment with CP or EMF triggered similar protein expression changes in sunflower shoots as illustrated by a similar pattern of upregulated and downregulated genes (clusters 1 and 3 in Fig. 4). This further supported the notion that radio-frequency radiation component of CP and EMF treatment of seeds could be the main cause of the changes in protein expression of the germinated sunflower shoots.

Although understanding about effects of CP on plant physiology is vague, several studies focused on radio-frequency EMF irradiation-induced gene expression regulation in plant cells were published previously (reviewed by^46^) and demonstrated that direct exposure to low power high frequency EMF radiation evokes changes in plant gene expression and modifies numerous metabolic activities (reactive oxygen species metabolism, Krebs cycle, pentose phosphate pathway, chlorophyll content, terpene emission, and gene expression)^46–50^. Further, it has been demonstrated that the response could occur not only in directly exposed tissues but could spread systemically to unaffected tissues through Ca^2+^ mediated signalling^51^.

Plant exposure to abiotic or biotic stress stimuli leads to activation of specific stress response pathways that results in accumulation of pathogenesis related proteins, chaperones implicated in protein stabilization, such as pathogenesis-related (PR) and heat shock proteins (HSP), and antioxidative enzymes involved in detoxification of reactive oxygen species^14,52^. It could be presumed that the CP or EMF treatment induced changes in seed phytohormone content might have seed-priming effect similar to results obtained by other seed-priming techniques^53^. Significant upregulation of the extracellular papain type cysteine proteinase family enzyme that participate in immune response in plants^54^ was detected upon the CP/EMF treatment. However, the role of the enzyme in the germinated sunflower seedlings is ambiguous as the treatment did not induce significant accumulation of PR or HSP proteins or other broad qualitative differences in protein expression pattern characteristic to abiotic stress response proteome^14^.

The consistent (low biological variance) low amplitude (-2.3 to 1.6, -2.6 to 3.9 and -3 to 2.7-fold for vacuum, CP and EMF treatment, respectively) differences in gene expression revealed by the proteome analysis imply that the treatment of seeds did not trigger distinct defense response or other stress induced developmental program in sunflower shoots, but rather predetermined a subtle modulation of plant metabolic processes that led to the observed phenotypic differences of the seedlings. Such gene expression changes are characteristic to low intensity stress (eustress) stimuli such as has been described for low intensity UV-B treatment that affects mainly the expression of genes that play role in the regulation of the cellular redox balance (enzymes involved in glutathione, pyridoxine, and phenolic metabolism) (reviewed by^55^). An increase in abundance of chloroplastic enzymes glutathione S-transferase and lipocalin, that are implicated in antioxidative function in chloroplasts^31,56,57^, was detected. In addition, a role of polyphenol oxidase in protection against low-level, chloroplastically derived oxidative stress has been recently proposed^58^. Since no differences in the expression of cytosolic redox balance regulating enzymes could be detected, it appears that the eustress-like response to the CP/EMF stimulus of the sunflower seedlings was mainly localized to chloroplasts.

Recent proteomic study on microwave (1.8 GHz) electromagnetic radiation effect on *Microcystis aeruginosa* algal cells grown in a bioreactor system revealed that the treatment downregulates accumulation of photosynthetic pathway proteins^50^ which is contradictory to our findings concerning a long-term effect of seed pre-treatment with EMF. Upregulated expression of enzymes involved in photosynthetic electron transfer (FNR1, plastocyanin, regulatory subunits O-2 and P-2 of photosystem II) or carbon fixation (rubisco subunit 1B, phosphoribulokinase, phosphoglycolate phosphatase) suggests that seed treatment resulted in a photosynthetic activity stimulating effect in the germinated sunflower shoots. This is further supported by the detected changes in differential expression of several proteoforms linked to PSBP2, the regulator of oxygen evolving complex activity in the PSII. In addition, several other chloroplastic or cytosolic proteins implicated in regulation of alternative photosynthetic pathways (ZKT protein^29^, thioredoxin M4^28^, ATP synthase^59^), and carbon metabolism (GAPC1^60^) were differentially expressed.

The CP or EMF treatment-specific differences were mostly limited to a slight variation in protein expression of proteins assigned to clusters 1 and 3 (Fig. 4). In addition, more pronounced qualitative differences were detected for two identified proteins of cluster 2, rubisco activase (RCA) and translationally-controlled tumor homolog proteins (TCTP). The RCA is the regulatory subunit of the PSII that regulates dark-light transitions under changing environmental conditions^61^. Further investigation would be required to understand the significance of this RCA proteoform in plant response to high-frequency radiation treatment. Another protein, TCTP, has been specifically upregulated by the EMF treatment. The protein is highly conserved among many eukaryotic organisms and is an important regulator of cellular growth in plants^62^, is involved in abiotic stresses response^63,64^ and plays crucial role in DNA repair^65^. The latter function of TCTP is important for cellular response to UV or ionizing radiation treatment but appears irrelevant to our experimental setup where non-ionizing EMF radiation was used. Therefore, EMF treatment-specific upregulation of TCTP is more likely a consequence of priming of stress response in seeds or regulation of cell growth.

The majority of the differentially expressed proteins identified in our study are involved in tightly linked network related to photosynthetic energy transfer, carbon fixation, carbohydrate metabolism or other chloroplastic and cytosolic processes implicated in the regulation of the photosynthetic activity (Fig. 5). The specific effect of the CP/EMF-treatment on the photosynthetic process is consistent with the finding that no significant protein expression differences could be detected in seedling roots. The later fact also supports a notion that the protein expression differences are not a consequence of treatment-induced difference in seed germination timing, as this could be expected to result in significant differences in root proteome, as well.

## Conclusion

We report for the first time that short treatments of sunflower seeds induce stressor specific pattern of changes in the content of seed phytohormones involved in the control of germination: vacuum treatment affected auxin/cytokinin balance; CP treatments substantially increased gibberellin content while other effects varied with treatment duration; EMF treatment was effective in decreasing abscisic acid content. Such a finding indicates that despite high resistance to environmental stresses in a dehydrated state, seeds rapidly respond even to short pre-sowing treatments with physical stressors on the level of phytohormone balance. Our results show that exposure of seeds to radio-frequency EMF or CP could induce a similar long-term effect on gene expression and the development of germinated plants suggesting that radio frequency radiation component of CP and EMF could be the main cause of the observed effect. The treatment has a moderate stimulating effect on expression of proteins mostly involved in photosynthetic pathways or their regulation and the protein expression differences are not related to defense or stress response priming in seeds.

The results of phytohormone balance and protein expression analysis provide an original insight into the molecular basis of plant phenotypic plasticity upon radio-frequency radiation treatment. These findings pave the way for further studies on seed germination physiology and regulation of photosynthetic activity in response to stressors.

## Methods

### Plant material

Seeds of the common sunflower confectionery variety ‘Nyķrségi fekete’ harvested in 2016 were received from the Institutes for Agricultural Research and Educational Farm, University of Debrecen (Hungary). Seeds were visually checked for quality, packed into plastic bags and transported for treatment with CP and EMF.

### Pre-sowing seed treatment with vacuum, CP and EMF

Seed treatments were carried out at the B. I. Stepanov Institute of Physics, NAS of Belarus (Minsk, Belarus). The equipment and conditions used for seed treatment have been described earlier in more detail^19^.

Seed treatment with radiofrequency (RF) EMF was carried out under the following experimental conditions: RF generator frequency − 5.28 MHz; root-mean-square value of magnetic *H* and electric *E* components of EMF strength were equal respectively 590 А/m (*В* ≈ 0.74 mT) and 12.7 kV/m; amplitude values H* = $\sqrt{2}\bar{H}$ and E* = $\sqrt{2}\bar{E}$ of 835 A/m (*В* ≈ 1 mT) and 17.96 kV/m, respectively. Packed seeds were placed in plastic bags on the container at the center of the induction coil and treatment was performed for 5, 10, and 15 minutes (these treatments are further abbreviated as EMF5, EMF10 and EMF15, respectively) at atmospheric pressure and room temperature.

The planar geometry reactor for seed treatment by CP consisted of two plane-parallel, water-cooled copper electrodes (120 mm diameter) placed in a stainless-steel vacuum chamber. The low-pressure capacitively coupled RF discharge in this reactor operated at 5.28 MHz in air (at a pressure of 200 Pa), and the specific power of 0.35 W/cm^3^ was applied. Seeds were evenly dispersed on the surface of an open, sterile Petri dish and placed on the grounded electrode before pumping air from the chamber. In every CP experiment, before plasma ignition between the electrodes, a pressure of 200 Pa (partial vacuum) was achieved by pumping air from the chamber for approximately 7 min. Thus “vacuum” treatment was used as an additional control in the CP experiments. Further CP treatment lasted for 2, 5, or 7 minutes (these treatments are abbreviated as CP2, CP5 and CP7, respectively).

Treatments for all experimental conditions were replicated three times. After treatment with CP and EMF seeds were stored in plastic bags at room temperature (20–22 °C) until the further investigation.

### Measurement of seed germination and seedling morphological parameters

Germination tests were started four days after the treatment both *in vitro* and in the substrate. For germination test *in vitro* seeds were evenly distributed on three layers of filter paper in 13.5 mm diameter plastic Petri dishes (three replicates of 30 seeds each) and watered with 6 mL distilled water. Petri dishes with seeds were placed in a climatic chamber KK 750 (Pol-Eko-Aparatura, Poland) with automatic control of moisture (60%), light, and temperature. Alternating light and temperature regimes were maintained in the chamber (darkness: 14 °C for 8 h; light: 21 °C for 16 h). Seeds were provided with additional water in a Petri dish, if necessary, to prevent drying. Germinated seeds (judged by the appearance of a visible 1-mm radicle) were counted daily until their number stopped increasing. For the germination tests in the substrate, the seeds were sown into plastic containers (12 × 18 × 30 cm) filled with peat substrate, placing the seeds in 0.5 cm depth from the substrate surface. Germination tests were replicated three times for all experimental conditions including control seeds (3 × 30 seeds, n = 30 for one replicate). Germinated seeds were counted daily as judged by the appearance of the top of green sprout from the surface of the substrate.

The germination results of each experimental replicate were analyzed using the application of Richards’ function^66^ for the analysis of germinating seed population^67^. The indices of germination kinetics derived from Richards plots were: V_i_ (%) – final germination percentage indicating seed viability, M_e_ (days) – median germination time (t_50%_) indicating the germination halftime of a seed lot or germination rate^67^.

The containers with grown seedlings were kept for 2 weeks in the climatic chamber with constant humidity (60%) and alternating light and temperature regimes (darkness: 14 °C for 8 h; light: 21 °C for 16 h). For morphometric analysis seedlings were carefully removed from containers, their roots washed to remove the substrate and wiped well with a moisture absorbent paper. Fresh weight and length of all seedlings and their parts (roots, shoots and leaves) was estimated.

### Phytohormone extraction from seeds and detection by HPLC analysis

For the extraction of plant hormones 1 g of seeds was ground and extracted in 5 mL of 85% methanol for 24 hours at 4 °C. The homogenate was centrifuged at 13500 × g for 5 min, the supernatant was collected and kept at −80 °C until HPLC analysis. Extractions were performed in triplicates.

Seed extracts were treated and analysed by a modified method of Bendokas *et al*.^68^ Plant hormones were separated and quantified using high performance liquid chromatography (HPLC). Agilent 1200 series HPLC system (Agilent Technologies Inc., USA) with a diode array detector and a reversed phase column (Spherisorb ODS2, 4 × 125 mm, Waters Corporation, USA) were used. Quaternary solvent (A 50% methanol, B 50% methanol, 1.2% acetic acid, C water, D methanol) gradient elution was used as follows: initial conditions 10% B, 60% C; 10.5 min 50% B, 15.75 min 50% B; 23 min 40% B, 60% D, 30 min 40% B, 60% D, and 32 min 10% B, 60% C. Gibberellins (GA3 and GA7) and ABA were detected at a wavelength of 254 nm, while auxins (IAA and IBA), Z and SA − at 280 nm. Peak positions of analytes were identified by the retention time, peak spiking and spectral properties. Hormone concentrations were valued via a linear regression equation of standard calibration curves. The analyses were performed in triplicate and the results were presented as mean ± standard error of mean.

### Sunflower seedling proteome analysis using two-dimensional electrophoresis

Seedlings grown from the control and vacuum, CP and EMF treated seeds were maintained in a climatic chamber as described above and the analysis was performed after 2 weeks of cultivation. Four and three biological repeats of protein samples from shoots and roots, respectively were prepared using phenol extraction and ammonium acetate precipitation, as described previously^69^. Internal standards were prepared from a pooled mixture of all protein extracts. Protein separation and detection was performed using a differential gel electrophoresis procedure as described previously^70^. Sample aliquots of 50 µg were labeled with Cy3 and Cy5 fluorescent dyes, and the internal standard was labeled with Cy2 dye (Lumiprobe, USA). For the preparative gel, 500 µg of unlabeled internal standard was mixed with 50 µg of Cy2 labeled internal standard. Isoelectric focusing was performed on 24 cm IPG strips with a linear gradient of pH 4–7 using Ettan IPGphor (GE Healthcare, USA). Further, the proteins were separated on 1-mm thick 10–16% polyacrylamide gradient gels using Ettan DALTsix (GE Healthcare, USA). Gels were scanned using a fluorescence scanner FLA 9000 (GE Healthcare, USA). Relative protein quantification was performed using DeCyder 2-D Differential Analysis Software, v.7.0 (GE Healthcare, USA).

Preparative gel was fixed in 50% methanol and 10% acetic acid. Protein spots were excised manually and subjected to protein digestion with trypsin, according to a method described previously^71^. Protein digests were loaded and desalted on a 100 μm × 20 mm Acclaim PepMap C18 trap column and separated on a 75 μm × 150 mm Acclaim PepMap C18 column using an Ultimate3000 RSLC system (Thermo-Scientific, USA), coupled to a Maxis G4 Q-TOF mass spectrometer detector with a Captive Spray nano-electrospray ionization source (Bruker Daltonics, Germany). Peptide identification was performed using the MASCOT server (Matrix Science, USA) against *Helianthus annuus* L., genome database v.1.0^25^. Threshold value for the identification of proteins was a Mascot score of >50 and at least 2 peptides.

Blast2GO software^72^ was used for the annotation and gene ontology analysis of the protein sequences identified with the NCBI Protein database. The obtained GO terms were summarized using the REVIGO server^73^, the *A. thaliana* database and the SimRel semantic similarity method with the level set at 0.7 value. *A. thaliana* homologues of the identified proteins were obtained by a search against TAIR10 gene models using the BLAST tool at the Sunflower Genome Database (https://sunflowergenome.org/blast/) and interactions were assessed using the String database with default settings^74^.

### Statistical data analysis

The Biological Variation Analysis module of the DeCyder software was used to match protein spots in biological repeats across different gels and ANOVA analysis was used to identify statistically significant (p ≤ 0.01) differences in protein abundance. Additionally, a threshold value of at least a 1.5-fold difference in protein abundance was used. Since CP treatments require application of a vacuum, the effect of CP treatment was compared to control and vacuum treated experimental groups.

Means of various parameters between the control and treatment groups were compared using Student’s t-tests for independent samples, as there was no reason for comparing different conditions of affected groups. The differences were considered to be statistically significant at p ≤ 0.05. The number of measured seeds or plants in the control and treatment groups varied from 17–24 (analysis of morphometric parameters) to 30 (germination tests and estimation of phytohormones content) for one replicate. Data are presented as means of 3 independent experiments ± standard error of mean.

## Supplementary information

Supplementary information
