# Supplementary material for: Treatment of Common Sunflower (Helianthus annus L.) Seeds with Radio-frequency Electromagnetic Field and Cold Plasma Induces Changes in Seed Phytohormone Balance, Seedling Development and Leaf Protein Expression
Source: Sci Rep. 2019 Apr 23;9:6437. doi: 10.1038/s41598-019-42893-5 (PMC6478675; doi:10.1038/s41598-019-42893-5)
Supplement: Supplementary file 1 — Supplementary information [file 41598_2019_42893_MOESM1_ESM.pdf]

Treatment of common sunflower (*Helianthus annus* L.) seeds with radio-frequency electromagnetic field and cold plasma induces changes in seed phytohormone balance, seedling development and leaf protein expression

Vida Mildažienė[1], Vesta Aleknavičiūtė[1], Rasa Žūkienė[1], Giedrė Paužaitė[1], Zita Naučienė[1], Irina Filatova[2], Veronika Lyushkevich[2], Perttu Haimi[3], Inga Tamošiūnė[3], Danas Baniulis[3]

[1] Faculty of Natural Sciences, Vytautas Magnus University, Kaunas, Lithuania
[2]B. I. Stepanov Institute of Physics, National Academy of Sciences of Belarus, Belarus [3]Institute of Horticulture, Lithuanian Research Centre for Agriculture and Forestry, Babtai, Kaunas reg., Lithuania

[*] **Correspondence:** vida.mildaziene@vdu.lt

Supporting information

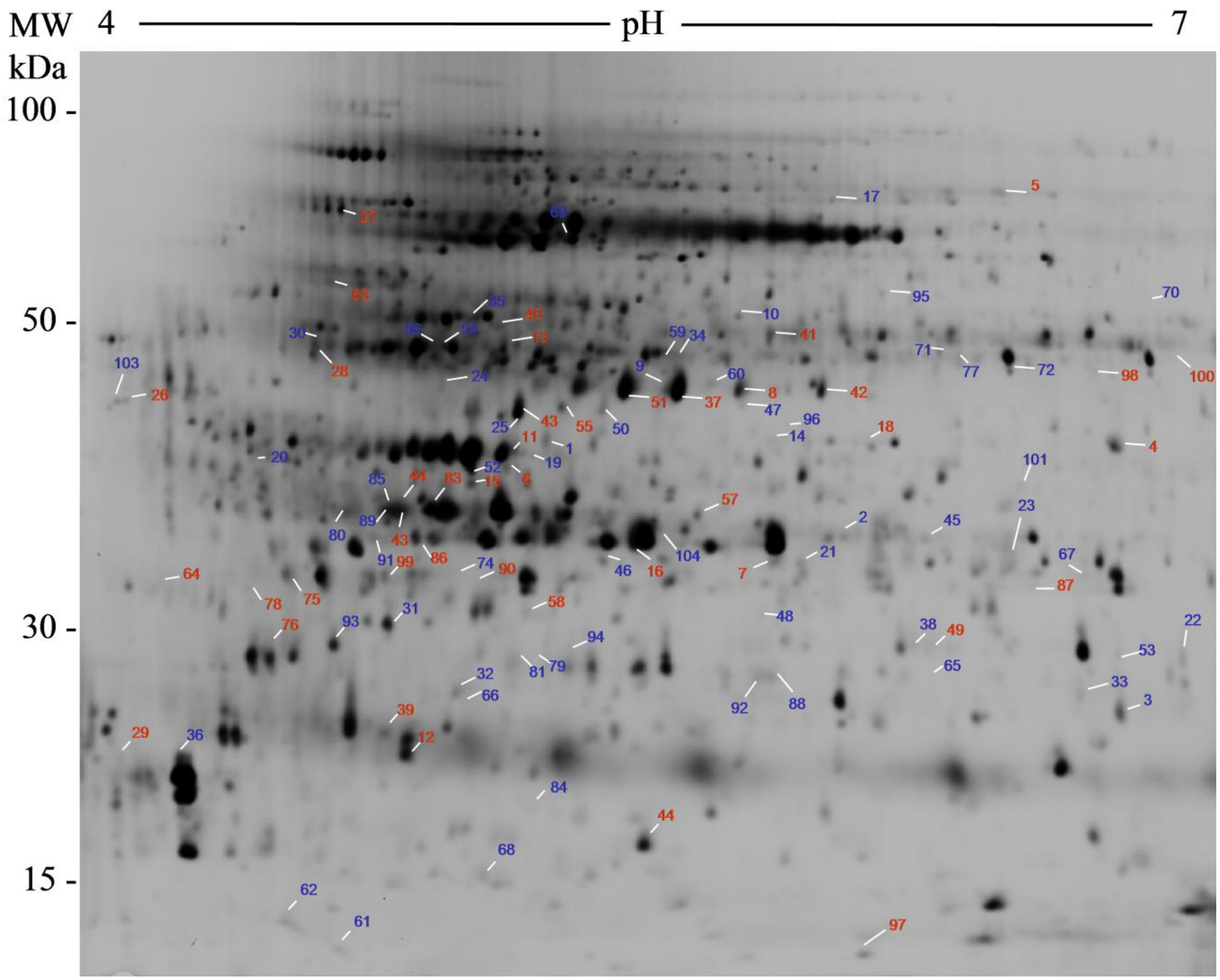

Figure S1. Two-dimensional electrophoresis gel of sunflower shoot protein samples. Results of the master gel including 50 μg pooled sample of total cell protein, separated on the acidic (pH 4–7) IPG strip (pH range is indicated at the top) and 10-16 % PAGE gel (molecular weight (MW) standard was not used on the gel and the tentative molecular weight indicated on the left was estimated based on MW of identified proteins) and visualized by scanning of Cy2 dye fluorescence are shown. Arrows indicate 104 spots differentially expressed upon vacuum, CP or EMF radiation. Unequivocally identified spots are labeled with red numbers.

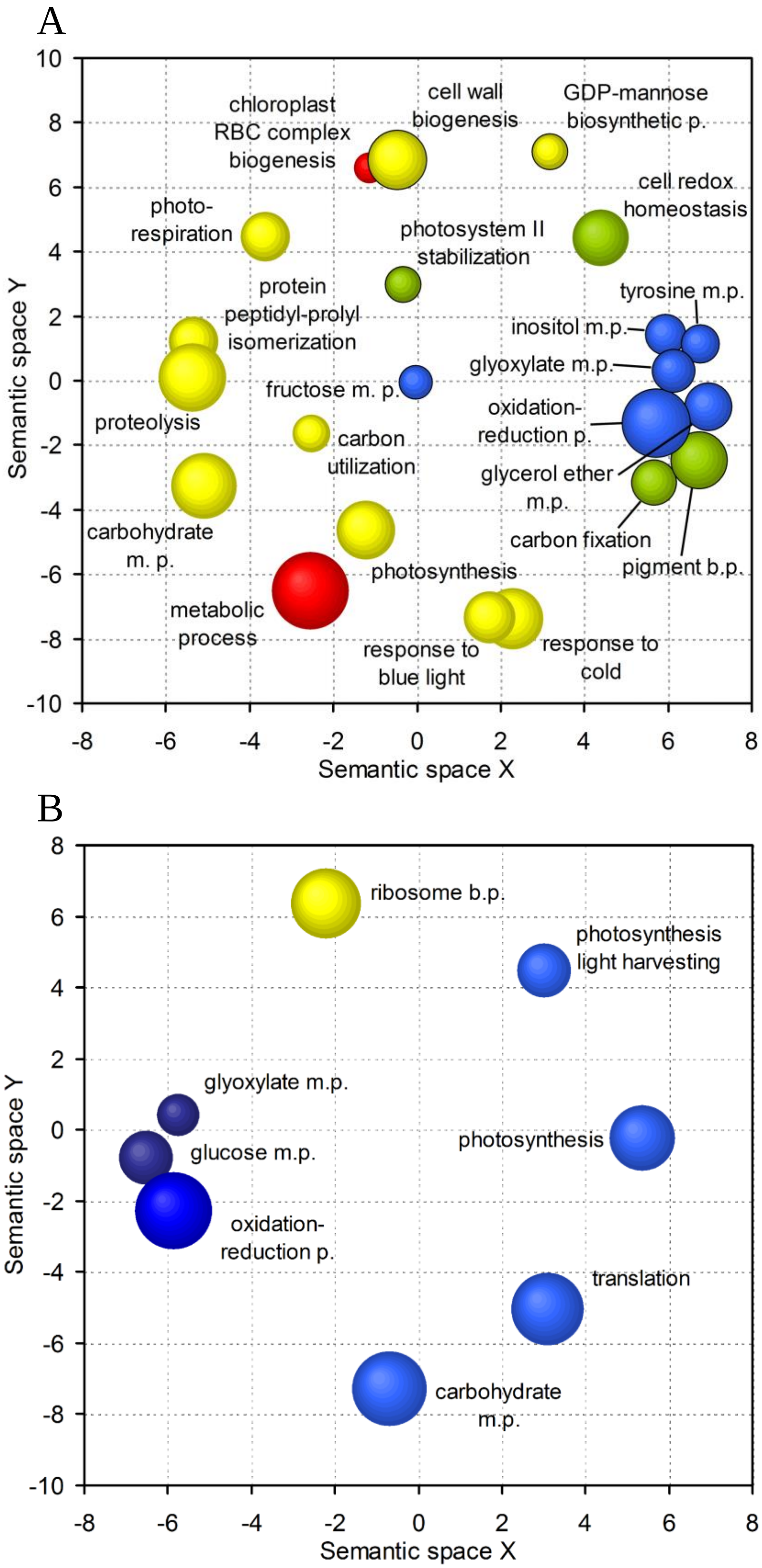

Figure S2. GO terms of biological process associated with the proteins of groups 1 and 4 (**A** and **B**, respectively) that were differentially expressed in sunflower shoots upon vacuum, CP or EMF treatment. The terms presented in semantic space were summarized using REVIGO algorithm. Size or cycles is proportional to frequency of GO terms. Color indicates uniqueness ((>0.4 – dark blue, >0.5 – blue, >0.7 – green, >0.8 – yellow, >0.9 – red).

Supplementary Table. Results of identification and annotation of 41 proteoforms differentially expressed upon vacuum, CP or EMF treatment.

Proteo-form No.	Peptide ID at *H. annuus* genome database	Mascot score	Num. of pep-tides	SC [%]	MW / pI	Protein ID[1]	TAIR accession ID	Protein name	Protein symbol
4	Ha412v1r1_12g035550	456	10	38	33.4 / 6.1	NP_569019.1	AT5G65730.1	Xyloglucan endo-trans-glucosylase/hydrolase 6	XTH6
5	Ha412v1r1_11g019250	108	3	7	67.5 / 6.0	NP_001306190.1	AT3G22270.1	Polyphenol oxidase, chloroplastic	PPO
6	Ha412v1r1_09g039480	1487	15	47	37.2 / 5.6	P85194.1	AT3G50820.1	Photosystem II subunit O-2	PSBO2
7	Ha412v1r1_06g011020	1587	14	52	28.1 / 8.7	P85189.1	AT2G30790.1	Photosystem II subunit P-2	PSBP2
8	Ha412v1r1_16g007260	426	10	38	39.0 / 6.9	O04977.1		Ferredoxin-$NADP^+$ reductase, leaf-type	FNR
11	Ha412v1r1_09g039480	1459	15	47	37.2 / 5.6	P85194.1	AT3G50820.1	Photosystem II subunit O-2	PSBO2
12	Ha412v1r1_00g072430	82	2	9	19.3 / 8.6	Q39746.1	AT5G38430.1	Rubisco small subunit 1B	RBCS1B
13	Ha412v1r1_03g036570	815	10	38	37.1 / 6.3	NP_174486.1	AT1G32060.1	Phosphoribulokinase	PRK
15	Ha412v1r1_15g046680	509	6	75	11.9 / 5.0	NP_190370.1	AT3G47860.1	Chloroplastic lipocalin	CHL
16	Ha412v1r1_14g033950	829	12	48	27.8 / 7.6	P85189.1	AT2G30790.1	Photosystem II subunit P-2	PSBP2
18	Ha412v1r1_04g040130	214	5	25	20.7 / 5.9	XP_015633714.1	AT2G45790.1	Phosphomannomutase	PMM
26	Ha412v1r1_16g010050	220	2	7	50.5 / 5.1	NP_568620.1	AT1G06260.1	Cysteine proteinase-like	CPLP
27	Ha412v1r1_15g007030	3008	41	78	56.0 / 4.7	NP_001310686.1	AT1G77510.1	Protein disulfide isomerase	PDI

Proteo-form No.	Peptide ID at *H. annuus* genome database	Mascot score	Num. of pep-tides	SC [%]	MW / pI	Protein ID[1]	TAIR accession ID	Protein name	Protein symbol
28	Ha412v1r1_01g006890	803	10	35	39.4 / 4.6	NP_564430.1	AT1G33811.1	GDSL esterase/lipase	GDSL-lipase
29	Ha412v1r1_05g027360	98	3	25	16.8 / 4.8	P00290.1	AT1G76100.1	Plastocyanin	PETE1
37	Ha412v1r1_15g026100	820	11	40	41.0 / 6.9	P10933.1	AT5G66190.2	Ferredoxin-NADP$^+$ reductase, leaf-type	FNR1
39	Ha412v1r1_11g011520	490	10	46	20.1 / 9.1	NP_564691.1	AT1G55480.1	ZKT protein	ZKT
40	Ha412v1r1_14g014920	697	14	55	33.9 / 5.5	XP_015616897.1		Rubisco activase	RCA
41	Ha412v1r1_00g062360	764	12	59	35.2 / 6.4	NP_190861.1	AT3G52930.1	Fructose-bisphosphate aldolase 8	FBA8
42	Ha412v1r1_16g007260	486	8	32	39.0 / 6.9	O04977.1		Ferredoxin-NADP$^+$ reductase, leaf-type	FNR1
43	Ha412v1r1_05g018150	226	5	23	33.8 / 5.3	NP_001119316.1	AT5G36790.3	Phosphoglycolate phosphatase	HAD
44	Ha412v1r1_13g032190	816	8	47	17.8 / 9.3	NP_188155.1	AT3G15360.1	Thioredoxin M4	TRX-M4
49	Ha412v1r1_05g003580	205	5	23	27.3 / 9.6	NP_179709.1	AT2G21130.1	Peptidyl-prolyl cis-trans isomerase	CYP19-2
51	Ha412v1r1_15g026100	1581	31	61	41.0 / 6.9	O04977.1	AT5G66190.2	Ferredoxin-NADP$^+$ reductase, leaf-type	FNR1
55	Ha412v1r1_07g001520	981	20	58	37.3 / 7.6	NP_564691.1	AT1G55480.1	ZKT protein	ZKT
57	Ha412v1r1_14g030950	562	12	56	28.5 / 7.1	XP_015641035.1		Chloroplastic glutathione S-transferase	GST
58	Ha412v1r1_06g011020	55	3	18	28.1 / 8.7	P85194.1	AT3G50820.1	Photosystem II subunit O-2	PSBO2
63	Ha412v1r1_09g023940	1204	17	46	52.5 / 5.4	Q7X9A0.1	AT2G39730.3	Rubisco activase	RCA

Proteo-form No.	Peptide ID at *H. annuus* genome database	Mascot score	Num. of pep-tides	SC [%]	MW / pI	Protein ID[1]	TAIR accession ID	Protein name	Protein symbol
64	Ha412v1r1_17g007380	147	3	21	18.9 / 4.4	Q9ZSW9.1	AT3G16640.1	Translationally-controlled tumor homolog	TCTP
75	Ha412v1r1_10g001650	661	11	43	27.0 / 7.7	NP_001312568.1	AT4G09650.1	Chloroplastic ATP synthase delta-subunit	ATPD
76	Ha412v1r1_07g004180	249	6	26	21.8 / 6.1	NP_001322828.1	AT1G64510.1	30S ribosomal protein S6	RPS6
78	Ha412v1r1_10g001650	768	12	39	27.0 / 7.7	NP_001312568.1	AT4G09650.1	Chloroplastic ATP synthase delta-subunit	ATPD
83	Ha412v1r1_03g028270	496	12	44	26.1 / 5.4	NP_001275214.1	AT1G16700.1	Alpha-helical feredoxin	NDUFS8
86	Ha412v1r1_03g021720	160	3	14	28.6 / 5.7	NP_178582.1	AT2G05070.1	Photosystem II LHC gene 2.2	LHCB2.2
87	Ha412v1r1_06g011020	309	5	27	28.1 / 8.7	P85194.1	AT3G50820.1	Photosystem II subunit O-2	PSBO2
90	Ha412v1r1_08g017490	279	5	14	26.7 / 6.4	NP_001234032.2	AT3G54890.4	Chlorophyll a-b binding protein of LHCI	LHCA1
97	Ha412v1r1_04g023910	328	3	30	14.6 / 9.9	NP_173755.1	AT1G23410.1	40S ribosomal protein S27a-1	RPS27AA
98	Ha412v1r1_12g028670	876	10	40	36.1 / 8.7	NP_564625.1	AT3G47520.1	Malate dehydrogenase	MDH
99	Ha412v1r1_06g011020	221	7	31	28.1 / 8.7	P85194.1	AT3G50820.1	Photosystem II subunit O-2	PSBO2
100	Ha412v1r1_08g002680	157	5	23	36.9 / 6.4	NP_187062.1	AT3G04120.1	Cytosolic glyceraldehyde-3-phosphate dehydrogenase 1	GAPC1
102	Ha412v1r1_06g011020	190	5	27	28.1 / 8.7	P85194.1	AT3G50820.1	Photosystem II subunit O-2	PSBO2

[1] NCBI RefSeq or UniProtKB/Swiss-Prot accession number provided at the NCBI Protein database. Abbreviations: SC – sequence coverage; MW – molecular weight; pI – isoelectric point.
